# Estimating the prevalence of schistosomiasis japonica in China: a serological approach

**DOI:** 10.1186/s40249-018-0443-2

**Published:** 2018-07-02

**Authors:** Xin-Yao Wang, Jing Xu, Song Zhao, Wei Li, Jian-Feng Zhang, Jian He, Ashley M. Swing, Kun Yang

**Affiliations:** 1 Jiangsu Institute of Parasitic Diseases, Yangxiang117#, Meiyuan, Wuxi, 214064 Jiangsu China; 2 Key Laboratory on Technology for Parasitic Disease Prevention and Control, Ministry of Health; Jiangsu Provincial Key Laboratory on the Molecular Biology of Parasites, Yangxiang117#, Meiyuan, Wuxi, 214064 Jiangsu China; 3 Public Health Research Center, Jiangnan University, Jiangsu, Sheng China; 4 National Institute of Parasitic Diseases, Chinese Center for Disease Control and Prevention, Shanghai, China

**Keywords:** Enzyme-linked immunosorbent assay (ELISA), Bayesian statistics, Schistosomiasis japonica, Sensitivity, Specificity, Estimated infection rate

## Abstract

**Background:**

The prevalence of schistosomiasis japonica has decreased significantly, and the responses changing from control to elimination in Jiangsu Province, P.R. China. How to estimate the change in prevalence of schistosomiasis using only serological data will be important and useful.

**Methods:**

We collected serum samples from 2011 to 2015 to build a serum bank from Dantu County of Jiangsu, China. Serum samples were detected by enzyme-linked immunosorbent assay (ELISA), the positive rate and optical density (OD) value were obtained. The Bayesian model including the prior information of sensitivity and specificity of ELISA was established, and the estimated infection rates were obtained for different years, genders and age groups.

**Results:**

There was no significant difference in the mean OD between different years and genders, but there was a significant difference between the different age groups. There were statistically significant differences in the positive rate for different years and age groups, but no significant difference at different genders. The estimated infection rate for the five years was 1.288, 1.456, 1.032, 1.485 and 1.358%, respectively. There was no significant difference between different years and between genders, but a significant difference between different age groups.

**Conclusions:**

The risk of schistosomiasis transmission in this area still exists, and risk monitoring of schistosomiasis should be strengthened.

**Electronic supplementary material:**

The online version of this article (10.1186/s40249-018-0443-2) contains supplementary material, which is available to authorized users.

## Multilingual abstract

Please see Additional file 1 for translation into in the five official working languages of the United Nations.

## Background

In tropical and subtropical regions, schistosomiasis remains as an important public health problem. Up to now, roughly 800 million people are at risk of schistosomiasis infection and more than 200 million people become infected [1, 2]. Over the past 50 years, the prevalence of schistosomiasis had decreased significantly, and the number of infections has greatly reduced in China [3–6]. Jiangsu Province is located in the lower reaches of the Yangtze River and has historically been one of the most affected regions of schistosomiasis in China. The cumulative number of patients was 253.07 million, and the cumulative area of the snail habitat was 1.47 billion square meters [7]. After 60 years of active and effective prevention and control, the prevalence of schistosomiasis finally fell below the government standard of 1% in 2010 [8, 9].

**Fig. 1:**
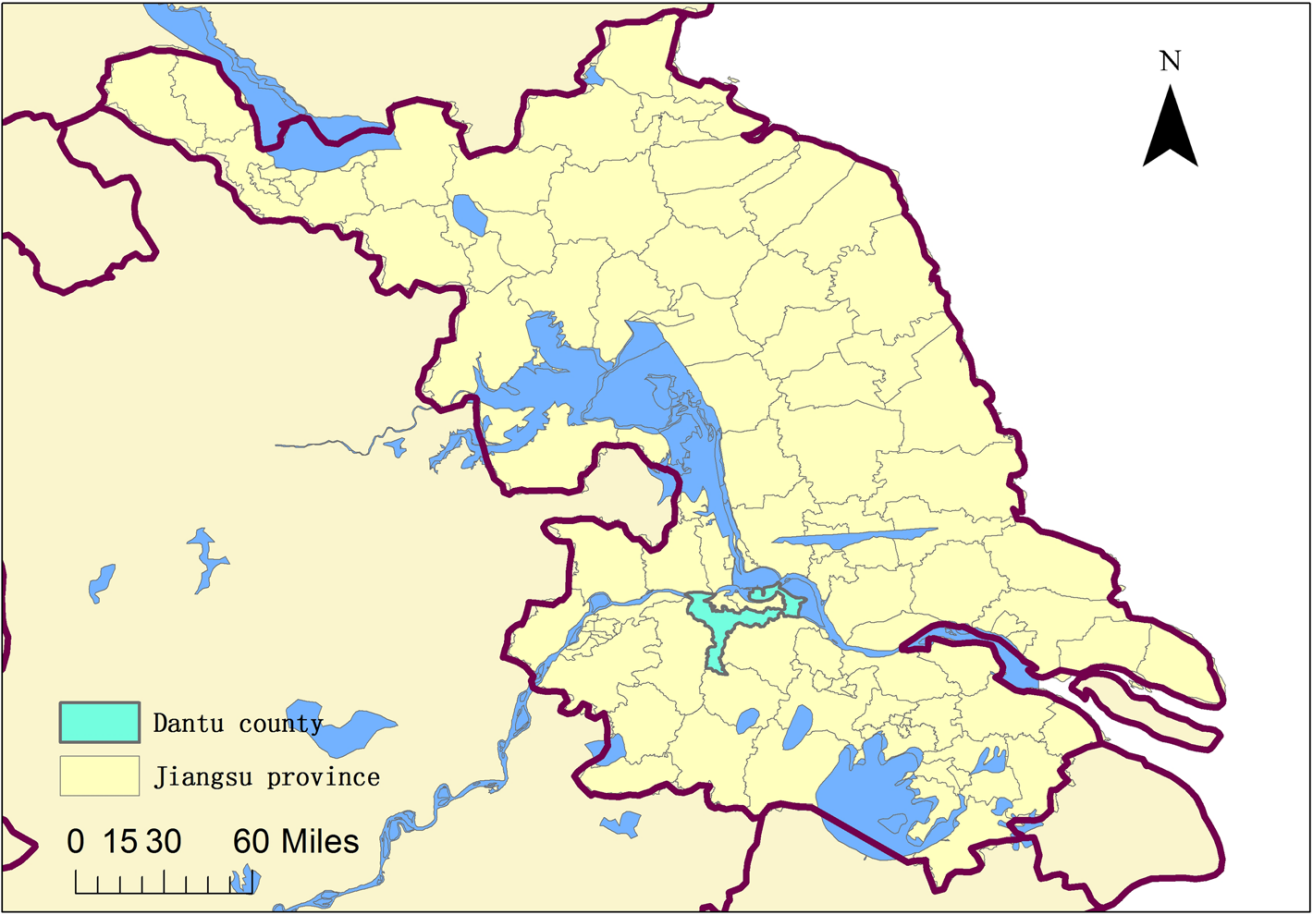
The location of Dantu County, Jiangsu Province, People’s Republic of China

Dantu County located in the southern aspect of Jiangsu Province and along the Yangtze River (Fig. 1), has been one of the most endemic counties (districts) with schistosomiasis in Jiangsu Province [10]. At the end of 2005, the cumulative number of all schistosomiasis patients was 4.85 million, and the area of living and the infected snail was 2.8634 and 0.551 km^2^, respectively. Acute schistosomiasis cases were also found every year from 2002 to 2005 [11]. The area of snail was 2.756 million square meters in 2011, and 766 000 square meters in 2015. The areas declined significantly, especially in the marshland and mountainous regions. The expanded chemotherapy was implemented on potential cases of schistosomiasis, and the number decreased from 159 to 45 in 2015 between 2011 and 2015.

In the early stages of schistosomiasis control, stool examination was improved by the implementation of the Kato-Katz thick smear method for schistosomiasis surveillance [12]. With the successful implementation of prevention and control project, the prevalence of schistosomiasis markedly decreased. But given the low sensitivity of the stool examination tests, the recent low levels of infection in the population have made an epidemiological investigation and estimation of the infection rate was very difficult [13]. With the rapid development of immunology and molecular biology techniques, some immunodiagnostic kits such as enzyme-linked immunosorbent assay (ELISA) or dipstick dye method have been developed and used in the field [13, 14]. In our previous study, ELISA test was proved with higher efficacy,and ELISA was selected for blood eximnation [15].

In this study, blood samples were collected from a serum bank for five consecutive years (2011–2015) and examined by ELISA. A serological approach was built to evaluate the effect of prevention and treatment.

## Methods

### Study region and samples selection

In this study, Wudun Village of Dantu County in Jiangsu Province is selected. The village is located on the middle island of Yangtze River. The village’s population is 2284 and has 809 households. The village is in an endemic area of schistosomiasis. By the end of 2017, the cumulative area of the snail habitat were 23.031 million square meters and the cumulative people of schistosomiasis were 2136 [16]. Blood samples from residents older than 6 years old were collected from October to November each year from 2011 to 2015. Other demographic information including name, gender and age was also collected at the same time. [17] All samples were stored in a − 70 °C frozen storage refrigerator in Jiangsu Institute of Preservation Diseases (JIPD). The standard process was used to prevent repeated freezing and thawing and ensure the quality of serum samples.

### Samples testing

In this study, the schistosomiasis antibody kit of ELISA that was used produced by Shenzhen Huakang Bio-Biomedical Engineering Co., Ltd. (Product batch number: 20160101). The value of optical density (OD) for ELISA was measured by a microplate reader. The OD value of all specimens was subtracted from the OD value of the blank control to obtain the true value of samples. A unified batch number of reagents was used to detect serum samples. Before the laboratory testing work, the person who participated in the investigation were trained. The training content included the use of ELISA reagents, read-out of results and OD value readings.

### Establishment of Bayesian model

The sensitivity and specificity of ELISA for the different age and different gender groups were calculated from a previously filed study [18]. Blood and stool samples were collected from 6 to 65-year-old residents. The Kato-Katz method was used to test stool samples, in which one stool sample was checked three times. ELISA method was used to test blood samples. We then used the Wilson interval algorithm to obtain a 95% confidence interval (*CI*) for sensitivity and specificity [19, 20].

In the process of Bayesian model construction, the prior distribution of sensitivity and specificity is assumed to be a beta (α, β) distribution [21, 22]. The beta distribution is a probability density distribution function between 0 and 1. Where π is the mean of the prior distribution of sensitivity or specificity, replaced by its prior central value. δ is the a priori standard deviation and is replaced by a quarter of its a priori range. The formula of α and β is:

$$ a=\pi \left[\frac{\left(1-\pi \right)\pi }{\delta^2}-1\right] $$

$$ \upbeta =\left(1\hbox{-} \uppi \right)\left[\frac{\left(1\hbox{-} \uppi \right)\uppi}{\updelta^2}\hbox{-} 1\right] $$

In addition, assuming a priori information without infection rate, a priori distribution is beta (1, 1). The variance ${\delta}_k^2$ , ${\updelta}_{\mathrm{j}}^2$ and ${\updelta}_{\mathrm{i}}^2$ of the normal distribution of age, gender and village random effects are subject to the no-information back-gamma distribution.

According to the prior distribution of sensitivity and specificity, Bayesian models were established only using the serological data to estimate the infection rate at different age and gender groups. The Bayesian model analyses were conducted in WinBUGS (Imperial College and MRC, London, UK), (http://www.mrc-bsu.cam.ac.uk/software/bugs/the-bugs-project-winbugs/) is neither nor allowed to have any missing value, so was subject to the following binomial distribution:

$$ {\mathrm{t}}_{\mathrm{k}}\sim \mathrm{Binomial}\left({\mathrm{p}}_{\mathrm{k},}{\mathrm{n}}_{\mathrm{k}}\right) $$

$$ {\mathrm{p}}_{\mathrm{k}}={\uppi}_{\mathrm{k}}{\mathrm{s}}_{\mathrm{j}}+\left(1\hbox{-} {\uppi}_{\mathrm{k}}\right)\left(1\hbox{-} {\mathrm{c}}_{\mathrm{j}}\right) $$

Where n_k_and t_k_ represent the population and positive rate of ELISA. The p_k_ and π_k_ represent infection rate of population and positive in the k age group, and the meanings of s_j_ and c_j_ were consistent with previous content.

The polynomial distribution was adjusted to:

$$ {\mathrm{p}}_{\mathrm{k}}={\uppi}_{\mathrm{k}}{\mathrm{s}}_{\mathrm{j}}\mathrm{z}\left[\mathrm{i}\right]+\left(1\hbox{-} {\uppi}_{\mathrm{k}}\mathrm{z}\left[\mathrm{i}\right]\right)\left(1\hbox{-} {\mathrm{c}}_{\mathrm{j}}\right) $$

$$ \mathrm{z}\left[\mathrm{i}\right]=\mathrm{dbern}\left({\updelta}_{\mathrm{z}}\right) $$

$$ {\updelta}_{\mathrm{z}}\sim \mathrm{beta}\left({\upalpha}_{\mathrm{z}},{\upbeta}_{\mathrm{z}}\right) $$

Where z [i] is the adjustment parameter for different villages. α_z_ and β_z_ is the prior distribution of the z [i].

Concerning the data structure, the information came from different layers, the first, second and third layer was studied year, gender and age group, respectively. The model was established as the following, and included the random effect at different layer [23]:

$$ \mathrm{logit}\left({\uppi}_{\mathrm{k}}\right)=\kern0.5em {\mathrm{uj}}_{\mathrm{k}} $$

$$ {\mathrm{uj}}_{\mathrm{k}}\sim \mathrm{normal}\left({\mathrm{ui}}_{\mathrm{j}},{\updelta}_{\mathrm{k}}^2\right) $$

$$ {\mathrm{u}\mathrm{i}}_{\mathrm{j}}\sim \mathrm{normal}\left({\mathrm{u}}_{\mathrm{i}},{\updelta}_{\mathrm{j}}^2\right) $$

$$ {\mathrm{u}}_{\mathrm{j}}\sim \mathrm{normal}\left(0,\kern0.5em {\updelta}_{\mathrm{i}}^2\right) $$

The uj_k_, ui_j_ and u_i_ represent the random effects of age, gender and year which were following the normal distribution, and were used to quantify the infection rate. The ${\updelta}_{\mathrm{k}}^2$, ${\updelta}_{\mathrm{j}}^2$ and ${\updelta}_{\mathrm{i}}^2$ are the variance of the corresponding variables.

According to the random effects of each gender ui_j_ and each year u_i_, the infection rate of each gender π_j_ and the infection rate of each year π_i_ can be calculated:

$$ {\uppi}_{\mathrm{j}}=\frac{\exp \left({\mathrm{u}\mathrm{i}}_{\mathrm{j}}\right)}{1+\exp \left({\mathrm{u}}_{\mathrm{i}}\right)} $$

$$ {\uppi}_{\mathrm{i}}=\frac{\exp \left({\mathrm{u}}_{\mathrm{i}}\right)}{1+\exp \left({\mathrm{u}}_{\mathrm{i}}\right)} $$

### Ethics statement

All studies described here were approved by the Ethics Review Committee of Jiangsu Institute of Parasitic Diseases, China (Permission number: JIPDERC2010008). The field studies did not involve endangered or protected species.

## Results

### Sensitivity and specificity of different gender and age groups

**Fig. 2:**
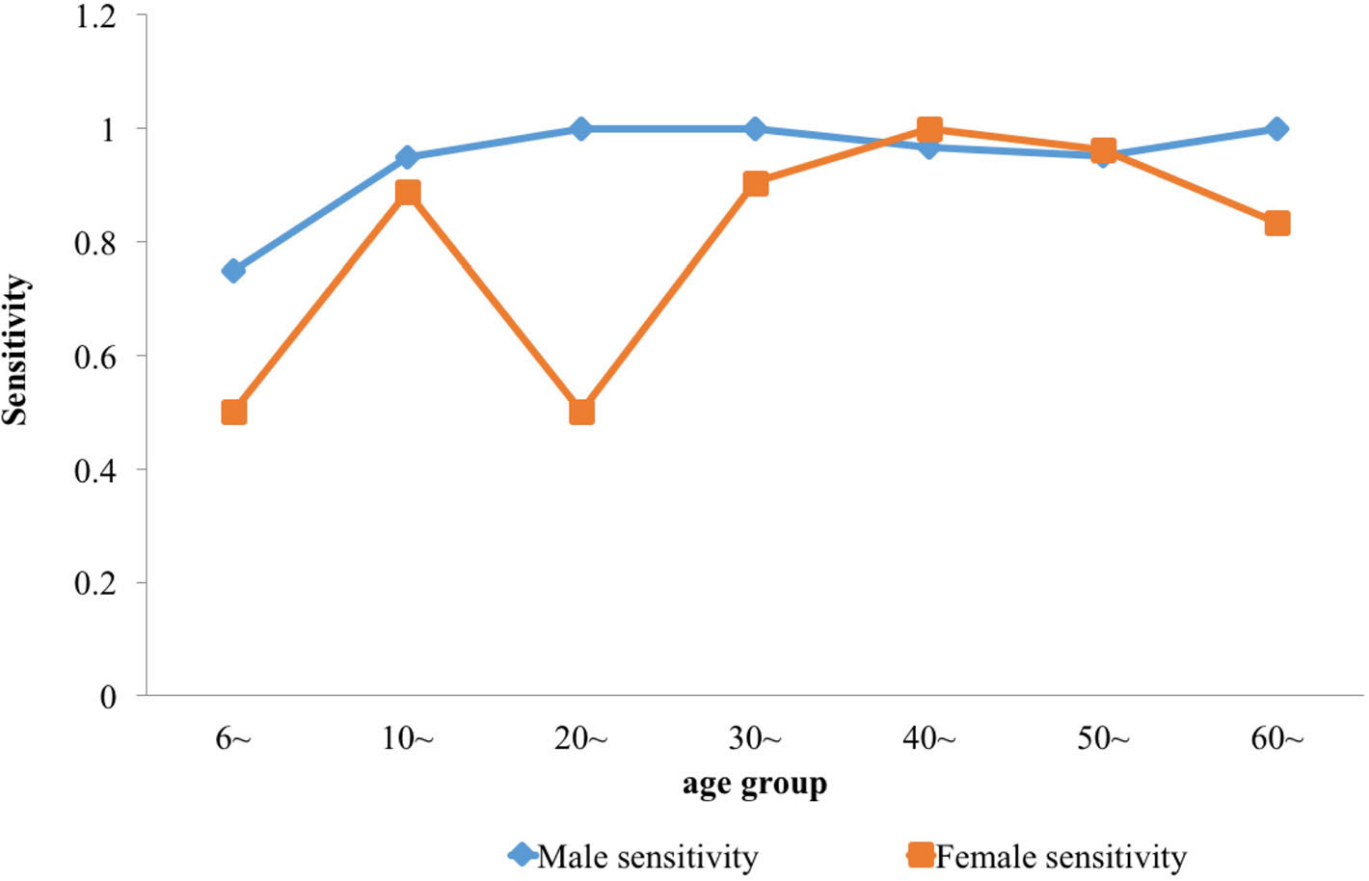
Sensitivity of males and females in different age groups in Dantu County, Jiangsu Province, People’s Republic of China

**Fig. 3:**
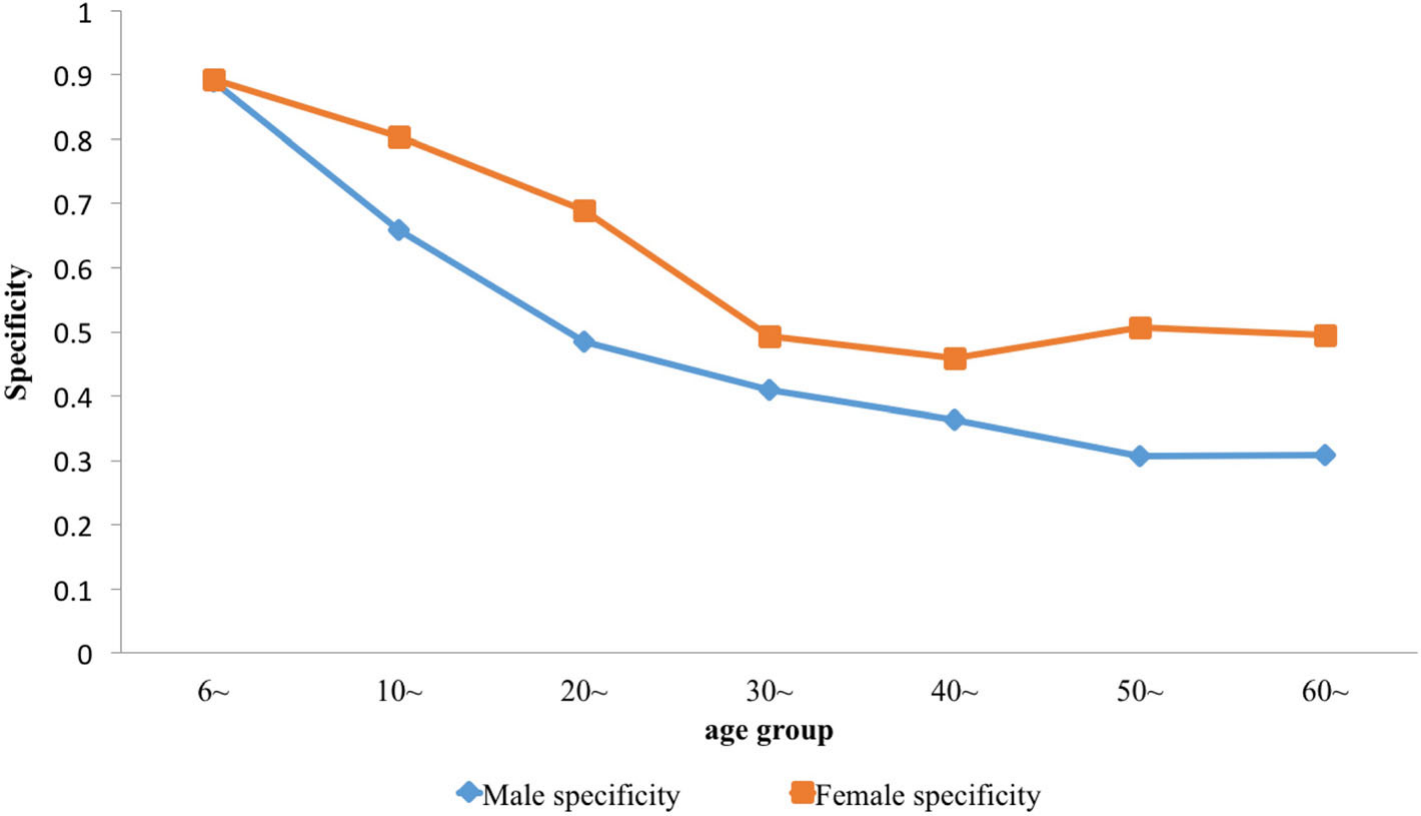
The specificity of males and females in different age groups in Dantu County, Jiangsu Province, People’s Republic of China

Table 1 shows the sensitivity and specificity of the serological test for different age and gender. On the whole, the sensitivity of both men and women increased with age. There was no significant difference in sensitivity (F = 2.426, *P* > 0.05) and specificity (F = 1.577, *P* > 0.05) between different genders. There was significant difference in sensitivity (F = 16.231, *P* < 0.01) and specificity (F = 7.727, *P* < 0.01) between different age groups. Among those 6 to 30 years old, the sensitivity of males and females improved as age increased. Its specificity showed a downward trend, and the specificity among males was lower than among females of the same age (Figs. 2 and 3).

**Table 1:** Prior distribution of Sensitivity and Specificity in ELISA of males and females in Dantu County, Jiangsu Province, People’s Republic of China

Age group	Sensitivity	Specificity	Sensitivity		Specificity	
	Mean (2.5% *CI*, 97.5% *CI*)	Mean (2.5% *CI*, 97.5% *CI*)	α	β	α	β
Males						
6~	72.81% (59.81,85.81%)	88.83% (87.60, 90.06%)	33.38841	12.468	2329.468	292.921
10~	94.15% (91.04, 97.26%)	69.51% (68.26, 70.75%)	213.5133	13.267	3801.042	1667.69
20~	91.95% (83.89, 100.00%)	51.05% (48.46, 53.63%)	41.06135	3.597	763.048	731.806
30~	99.30% (98.60, 100.00%)	41.19% (39.61, 42.76%)	562.4636	3.965	1608.251	2296.693
40~	96.37% (94.91, 97.83%)	35.51% (34.28, 36.73%)	631.6588	23.793	2166.815	3936.029
50~	94.71% (92.59, 96.82%)	30.61% (29.49, 31.73%)	423.7224	23.691	2072.924	4699.124
60~	98.96% (97.91, 100.00%)	31.83% (30.23, 33.42%)	373.8274	3.948	1085.359	2325.038
Females						
6~	50% (29.93, 70.07%)	89.51% (88.07, 90.95%)	11.91296	11.913	1620.368	189.897
10~	87.3% (80.74, 93.85%)	81.91% (80.82, 82.99%)	89.25677	12.991	4123.763	911.049
20~	94.33% (88.65, 100.00%)	69.91% (67.86, 71.95%)	61.76833	3.716	1405.945	605.277
30~	89.75% (85.75, 93.75%)	49.31% (47.92, 50.69%)	205.5135	23.471	2569.355	2641.79
40~	99.37% (98.74, 100.00%)	46.31% (45.21, 47.41%)	625.9521	3.969	3805.965	4412.486
50~	95.49% (93.07, 97.90%)	50.86% (49.69, 52.03%)	281.373	13.305	3713.784	3588.19
60~	94.33% (88.65, 100.00%)	55.04% (53.28, 56.80%)	61.76833	3.716	1758.255	1436.249

### The prior distribution of sensitivity and specificity

The sensitivity and specificity among males were between (0.7500, 1.0000) and (0.3059, 0.8898), and the sensitivity and specificity among females were between (0.000, 1.0000) and (0.4631, 0.8960). The 95% *CI* for sensitivity and specificity was obtained using the Wilson interval algorithm, and a prior distribution of sensitivity and specificity was obtained for different age groups and gender (Table 1).

#### Results of serological testing

**Fig. 4:**
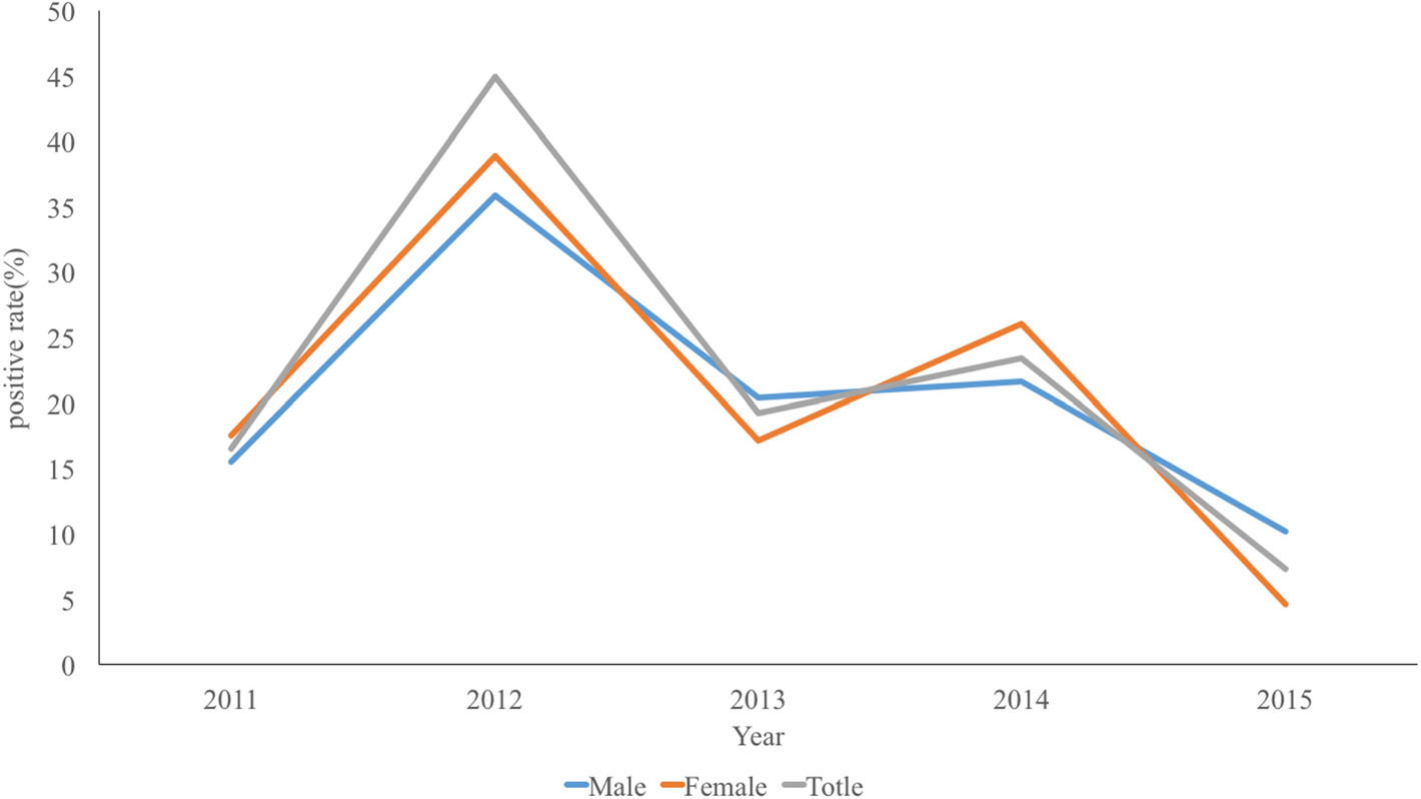
The positive rate of ELISA from 2011 to 2015 in Dantu County, Jiangsu Province, People’s Republic of China

In this study, 2180 blood samples were collected from 2011 to 2015, including 1132 samples from males and 1048 samples from females. The highest positive infection rate was 37.38% in 2012, and the lowest rate was 7.36% in 2015 (Table 2). The positive rate decreased year by year since 2012 (Fig. 4). The number of samples from males in 2011 was less than females in 2015, but the number of samples from males were greater than females in 2012, 2013 and 2014. The male positive rate from serology testing was lower than that of females in 2011, 2012 and 2014, but the male positive rate of serology test was higher than female in 2013 and 2015 (Fig. 4). The positive rate increased in the overall population as age increased from 40 years of age. The highest positive rate was among those 60 years old and older. There were statistically significant differences in the positive rate of serological tests at the monitoring point for all five consecutive years (*P* = 0.0001) and age groups (*P* = 0.0001). There was no significant difference in the positive rate of serology between different gender (*P* = 0.79).

**Table 2:** The positive rate of ELISA from2011 to 2015 in Dantan County, Jiangsu province, People’s Republic of China

Year	Gender	The positive	The negative	Positive rate(%)	Total
2011	Male	26	141	15.569	167
	Female	34	160	17.526	194
	Total	60	301	16.621	361
2012	Male	75	134	35.885	209
	Female	79	124	38.916	203
	Total	154	258	37.379	412
2013	Male	42	164	20.388	206
	Female	32	154	17.204	186
	Total	74	318	18.878	392
2014	Male	66	239	21.639	305
	Female	54	153	26.087	207
	Total	120	392	23.438	512
2015	Male	25	220	10.204	245
	Female	12	246	4.651	258
	Total	37	466	7.356	503

**Fig. 5:**
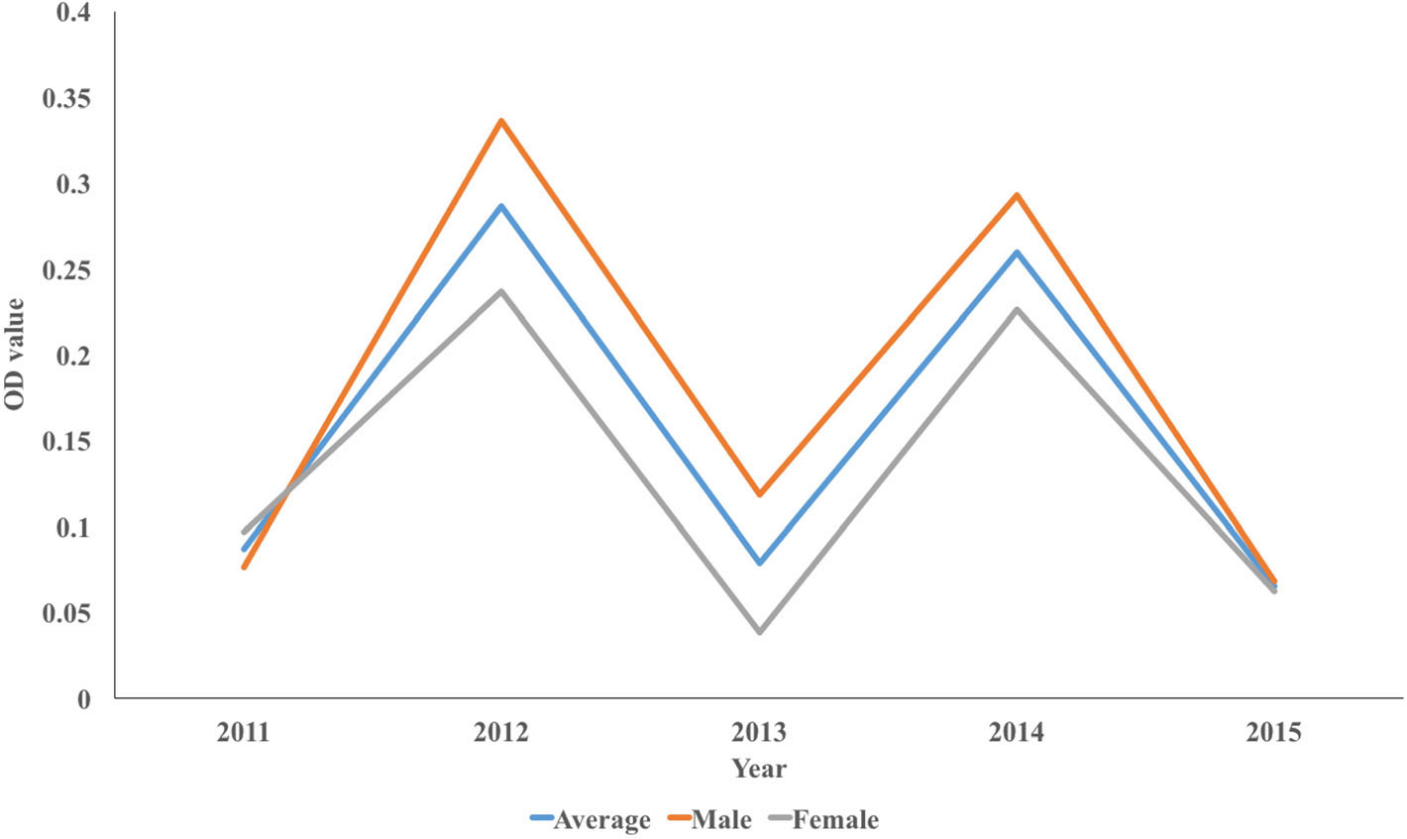
The OD value of ELISA from 2011 to 2015 in Dantu County, Jiangsu Province, People’s Republic of China

**Fig. 6:**
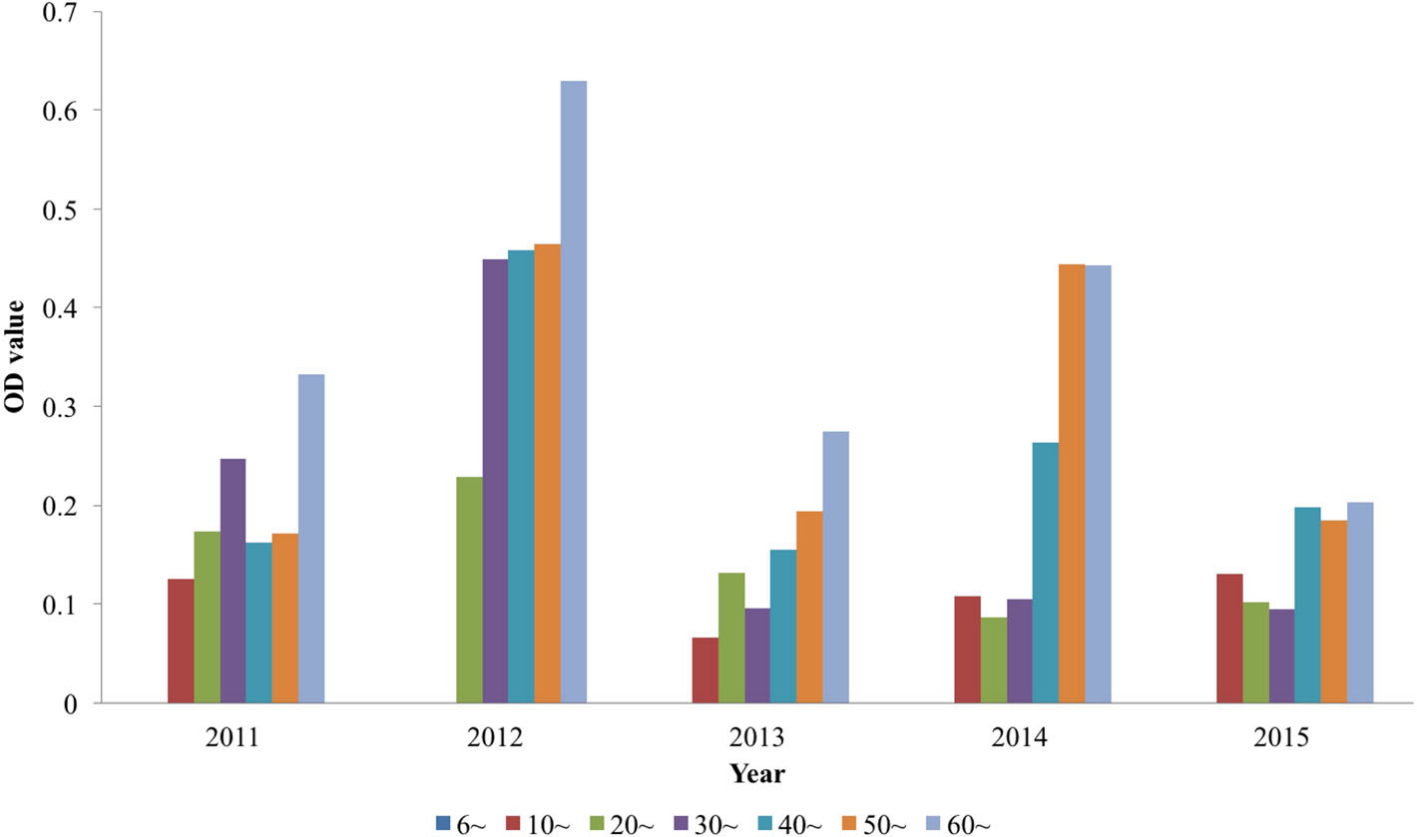
The average OD of the age groups from 2011 to 2015 in of Dantu County, Jiangsu Province, People’s Republic of China

The average OD value for 2011–2015 was 0.087, 0.287, 0.078, 0.260 and 0.065 from 2011 to 2015, with the highest in 2012 and the lowest in 2015. The OD values were similar between male and female groups (Fig. 5) with no significant difference (*P* = 0.113). The OD value gradually increased with age, and the average OD was the largest among those more than 60 years of age (Fig. 6). This difference was statistically significant (*P* = 0.0001), showing the OD value was positively correlated with age, with a correlation coefficient of 0.995 (*P* < 0.001). There was no significant difference in mean OD between different study years (*P* = 0.488).

### Estimating the infection rate of the population in monitoring points

**Fig. 7:**
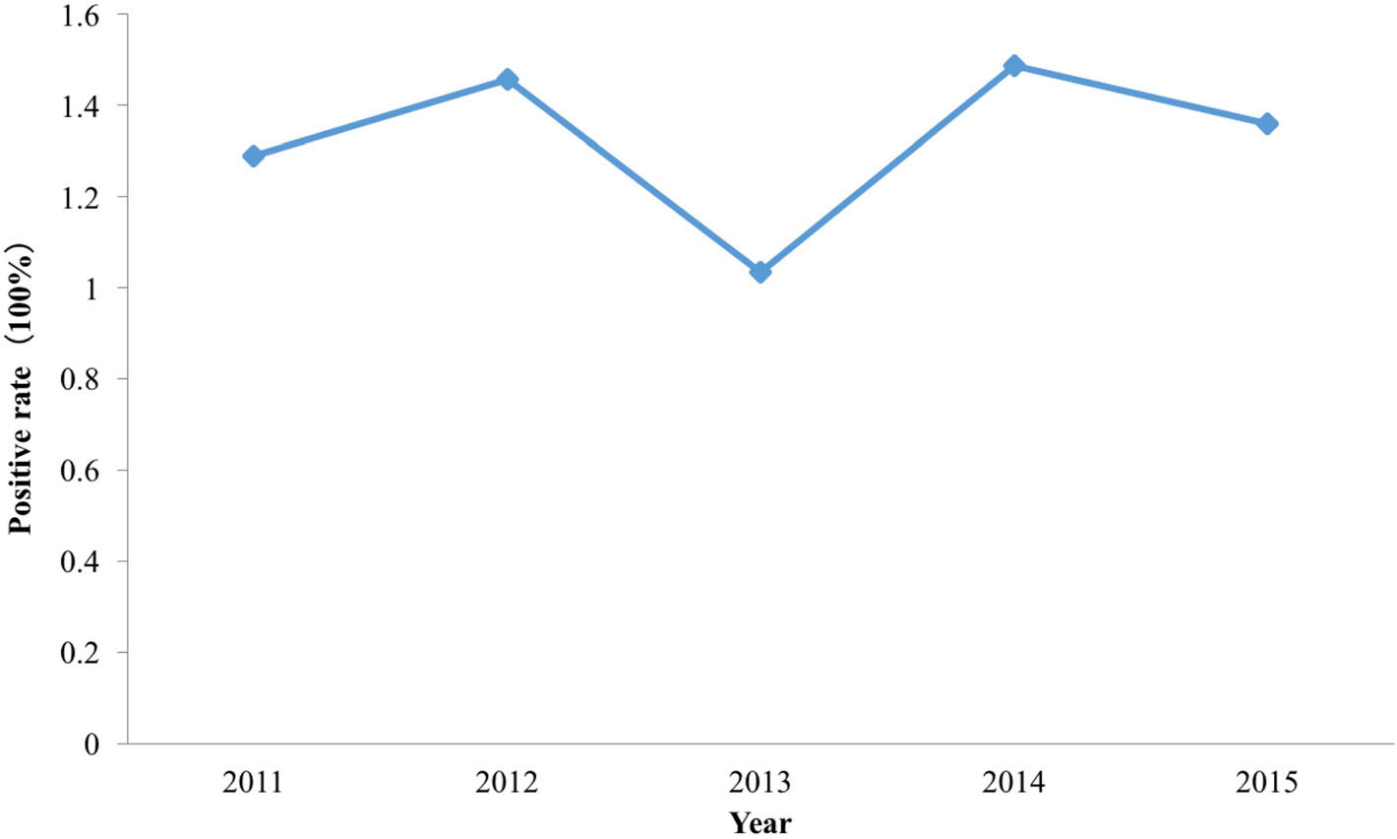
Estimation of infection rate of schistosomiasis from 2011 to 2015 in Dantu County, Jiangsu Province, People’s Republic of China

Table 3 shows the estimating infection rate for different genders and age groups. The infection rate from 2011 to 2015 was 1.288, 1.456, 1.032, 1.485 and 1.358%, with the highest in 2014 and lowest in 2013 (Fig. 7). However, there was no significant difference between different study years (*P* = 0.998). There was also no significant difference between different gender groups (*P* = 0.969), but there was a significant difference between different age groups (*P* < 0.05).

**Table 3:** The estimate infection rate of the population based on schistosomiasis Bayesian model of Dantu County, Jiangsu Province, People’s Republic of China

Year	Gender	Age group	Infection rate of age groups (%)	Infection rate of gender group (%)	Infection rate of years (%)
2011	Male	10~	1.924	1.043	1.288
		30~	1.92		
		40~	1.96		
		50~	1.922		
		60~	1.945		
	Female	10~	2.021	1.092	
		20~	2.004		
		40~	2.085		
		50~	2.07		
		60~	2.046		
2012	Male	20~	2.413	1.273	1.456
		30~	2.429		
		40~	2.38		
		50~	2.456		
		60~	2.41		
	Female	10~	2.701	1.422	
		30~	2.741		
		40~	2.705		
		50~	2.7		
		60~	2.662		
2013	Male	20~	1.391	0.778	1.032
		30~	1.422		
		40~	1.368		
		50~	1.367		
		60~	1.423		
	Female	20~	0.766	0.418	
		40~	0.796		
		50~	0.783		
		60~	0.776		
2014	Male	10~	2.959	1.577	1.485
		30~	2.945		
		40~	2.939		
		50~	2.912		
		60~	2.993		
	Female	10~	2.674	1.438	
		40~	2.704		
		50~	2.702		
		60~	2.713		
2015	Male	40~	2.207	1.154	1.358
		50~	2.272		
		60~	2.166		
	Female	10~	2.679	1.433	
		40~	2.795		
		50~	2.769		
		60~	2.687

## Discussion

According to this national report of schistosomiasis control, the schistosomiasis control processes are changing from working to keep schistosomiasis under control to interrupting transmission, with the final aim of elimination in China [24]. Previous studies indicated that there was a serious misdiagnosis of the pathogenicity method at low levels of prevalence [25–27]. In recent years, the Bayesian estimation model has been used to estimate the true infection rate without a gold standard [28–30]. So, we explored the use of a serological approach based on consecutive five years of serum library samples to estimate the true prevalence and evaluate the control effectiveness of schistosomiasis japonica. The five-year serum pool was used and no stool samples were collected. It was feasible to use the serum results to estimate the infection rate based on the published literature. Therefore, the infection rate was estimated using five consecutive sera samples. In the process of model building, the sensitivity and specificity of different age groups and gender were included, and then the corresponding parameter values were obtained from field investigation except for Delphi method [31]. This method differed from other detection methods in that there was no leak detection of stool test, and there was not too much false-positive serum to estimate the population infection rate. This approach can significantly improve the accuracy of estimations of disease prevalence and can reduce selection bias and information bias.

The data structure was nested with differing layers: the first layer was studied year, the second layer was the gender group from a different study year, and the third layer was the age group from a different gender group. In order to reflect this data structure, Bayesian hierarchical modelling was used to estimate the infection rates for the different layers, namely the study years, gender and age groups. There is some limitation in using the techniques. First, the sample size which the study calculates specificity and sensitivity may be low, especially some age group have this condition. Secondly, in general, Bayesian estimation model, higher specificity and sensitivity are needed to improve the accuracy of estimation. However, the specificity and sensitivity of some groups in this study are low, which have some impact on the results.

The change in the trend of estimated infection rate appeared similar to the serological detection rate, with increases in trend from 2011 to 2012 and 2013 to 2014, and decreases in trend from 2012 to 2013 and 2014 to 2015. However, there was a significant difference for infection rate from serologic testing (*P* = 0.0001), but no significant difference for the estimated infection rate (*P* = 0.998). This suggests that the population of Dantu County in the past five years has had no significant changes. Previous studies have shown that serological tests are difficult to distinguish between the current disease and the previous infection. When the human body is infected with schistosomiasis, the antibody level increases rapidly, then antibodies decrease significantly 2 months after chemotherapy. Antibody levels were not significantly reduced 2 to 8 months after chemotherapy [32].

In this study, the reagents for ELISA came from the same batch, and the reaction conditions were consistent so the OD value can be directly compared. There was no significant difference in mean OD value between different study years, suggesting that the antibody titer did not change throughout the years. The Bayesian model evaluates rate as a whole and does not take into account the individual condition. It is consistent with the OD value. The OD value can be used to assess the change of the epidemic, which is more reliable than the serum positive rate. The lack of significant infection rate differences between genders may be due to the fact that infection opportunities are the same across gender, and there is no difference in the overall level of antibodies between genders. There were significantly different between different age group at the estimated infection rates and the OD value serum samples. This may be due to differential exposure opportunities, given young people rarely have contact with water and other risk factors [33].

Previous studies have shown that this method can be used to monitor the work, and calculate the population infection rate. The main purpose of this study is to evaluate the epidemic situation of schistosomiasis by using the results of previous studies [34, 35]. We found that the risk of schistosomiasis transmission in this area still existed in 2011–2015. The risk monitoring of schistosomiasis needs to be strengthened, and the prevention and control work needs to be further enhanced, certain aspects of disease control can be improved, such as expanding to a more comprehensive management of the snail environment and human interactions with such areas, implementing surveillance of infection among livestock and poultry, improving stool treatment processes, and applying pharmaceutical interventions upon snail populations. Additionally, active and passive monitoring need to be combined to provide timely detection of local or imported schistosomiasis epidemics [24].

## Conclusions

The risk of schistosomiasis transmission in this area still exists, and risk monitoring of schistosomiasis should be strengthened. Jiangsu Province remains in strict accordance with the National Schistosomiasis Monitoring Program (2014 version) requirements, and carefully carries out regular monitoring of schistosomiasis [36] with the goal of providing timely detection and treatment in the event of an epidemic, and strive to for early detection, early treatment, and early control. Jiangsu Province has seen much success in the control of schistosomiasis across the province [37]. However, there remains the need to further strengthen the monitoring of schistosomiasis in the region in order to fully actualize the goal of schistosomiasis prevention and elimination.

## Additional file

Additional file 1: Multilingual abstract in the five official working languages of the United Nations. (PDF 203 kb)

## Abbreviations

*CI*: Confidence interval
ELISA: Enzyme-linked immunosorbent assay
OD: Optical density
