# Supplementary material for: Estimating the prevalence of schistosomiasis japonica in China: a serological approach
Source: Infect Dis Poverty. 2018 Jul 2;7:62. doi: 10.1186/s40249-018-0443-2 (PMC6027568; doi:10.1186/s40249-018-0443-2)
Supplement: Supplementary file 1 — Multilingual abstract in the five official working languages of the United Nations. (PDF 203 kb) [file 40249_2018_443_MOESM1_ESM.pdf]

Translation of the abstract into the five official working languages of the United Nations

وأُجريت عدة دراسات استقصائية لتقدير مدى انتشار الإصابة بفيروس داء البلهارسيات اليابانية في مقاطعة دانتو بجيانغسو، الصين

شين ياو وانغ، وجينغ شو، وسونغ تشاو، ووي لي، وجيان-فنغ تشانغ، وجيان هي،واشلي، وإم سوينغ، وكون يانغ

الملخص
الخلفية: انخفض بشكل كبير انتشارداء البلهارسيات اليابانيةوتبدلت الاستجابة من السيطرة على المرض إلى القضاء على المرض في مقاطعة جيانغسو، جمهورية الصين الشعبية. إن تقدير التغير في انتشار داء البلهارسيات باستخدام البيانات المصلية فقط سيكون مهماً ومفيداً.
الطرق: جمعنا عينات من مصل الدم من عام 2011 حتى عام 2015 لتأسيس بنك مصل الدم من مقاطعة دانتو بجيانغسو، الصين. تم الكشف عن عينات من المصل عن طريق مقايسة الامتصاص المناعي المرتبط بالإنزيم (ELISA) ، وتم الحصول على المعدل الموجب وقيمة الكثافة البصرية (OD). تم إنشاء نموذج بايزي بما في ذلك معلومات الحساسية السابقة وخصوصية مقايسة الامتصاص المناعي المرتبط بالإنزيم (ELISA) ، وتم الحصول على معدلات الإصابة التقديرية لسنوات مختلفة ، والجنسين والفئات العمرية.
النتائج: لم يكن هناك فرق كبير في متوسط قيمة الكثافة البصرية(OD) بين الجنسين، والسنوات المختلفة، لكن كان هناك فرق واضح بين مجموعات الأعمار المختلفة. كانت هناك فروق ذات دلالة إحصائية في المعدل الإيجابي لمختلف السنوات والفئات العمرية ، ولكن لا يوجد فرق كبير بين الجنسين. كان معدل الإصابة المقدر لمدة خمس سنوات 1.288 ٪ ، 1.456 ٪ ، 1.032 ٪ ، 1.485 ٪ و 1.358 ٪ على التوالي. لم يكن هناك فرق كبير بين السنوات المختلفة والجنسين، لكن يوجد فرق واضح بين مجموعات الأعمار المختلفة.
الاستنتاجات : مازال خطر انتقال عدوى البلهارسيات اليابانية متواجداً، وينبغي تعزيز رصد مخاطر داء البلهارسيات.

Translated from English version into Arabic by Dima shs and Free Bird, through

TRANSLATORS WITHOUT BORDERS

基于血清学方法的江苏省丹徒区日本血吸虫感染率估算研究

王鑫瑶，许静，赵松，李伟，张键锋，何健，Ashley M. Swing，杨坤

摘要
背景：江苏省血吸虫病疫情显著下降，逐渐从血吸虫病传播控制走向消除。如何单纯使用血清学数据估算血吸虫病流行状况，对于血吸虫病防控非常重要和有用。
方法：采集 2011 年至 2015 年丹徒区流行区血清样本，建立血清库。利用酶联免疫吸附试验（ELISA）检测，获得血清学检测阳性率和平均光密度（OD）值。建立含 ELISA 灵敏度和特异度先验信息的贝叶斯模型，并估算不同年份、性别和年龄组的感染率。
结果：不同年份和性别组平均 OD 值无显着性差异，但不同年龄组之间存在显着性差异差异。不同年份和年龄组阳性率存在显著性差异，但不同性别间无显著性差异。丹徒区连续 5 年的估算感染率分别为 1.288%，1.456%，1.032%，1.485%和 1.358%。不同年份和性别组的估算感染率无显着性差异，不同年龄组存在显着性差异。
结论：丹徒区血吸虫病传播风险依然存在，应加强血吸虫病风险监测。

Translated from English version into Chinese by Xin-Yao Wang and Kun Yang

Approche sérologique pour estimer la prévalence de Schistosoma japonica dans la circonscription chinoise de Dantu, province du Jiangsu

Xin-Yao Wang, Jing Xu, Song Zhao, Wei Li, Jian-Feng Zhang, Jian He, Ashley M. Swing et Kun Yang

Résumé

Contexte: La prévalence de *Schistosoma japonica* a significativement diminué et les réponses passent du contrôle à l'élimination dans la province du Jiangsu, en République populaire de Chine. Il va être important et utile de savoir comment estimer l'évolution de la prévalence de la schistosomiase sur la seule base des données de sérologie.

Méthodes:Nous avons recueilli des échantillons de sérum entre 2011 et 2015 dans la circonscription de Dantu, dans la province chinoise du Jiangsu, afin de constituer une base de sérums. Les échantillons de sérum ont été examinés par immunosorption enzymatique (ELISA) afin d'obtenir le taux d'échantillons positifs et la densité optique (DO). Le modèle bayesien, contenant les informations antérieures de sensibilité et de spécificité du test ELISA, a été créé et les taux d'infestation estimés ont été obtenus pour différentes années, les deux sexes et différents groupes d'âge.

Résultats: Aucune différence n'est apparue dans la densité optique moyenne entre les différentes années et les deux sexes ; en revanche, la différence est significative entre les différents groupes d'âge. Il y a eu des différences statistiquement significatives dans le taux d'échantillons positifs pour différentes années et différents groupes d'âge, mais pas entre les deux sexes. Le taux d'infestation estimé pour les cinq années était de 1,288 %, 1,456 %, 1,032 %, 1,485 % et 1,358 %, respectivement. Il n'y a pas eu de différence significative entre les années et entre les sexes mais bien entre les différents groupes d'âge.

Conclusions: Il existe encore un risque de transmission de la schistosomiase dans cette zone et la surveillance du risque doit être renforcée.

Translated from English version into French by Iris Soliman and Suzanne Assenat, through

TRANSLATORS WITHOUT BORDERS

Серологический подход к оценке распространённости японского шистосомоза в районе Даньту провинции Цзянсу, Китай

Синь-Яо Ван, Цзин Сюй, Сон Чжао, Вэй Ли, Цзянь-Фэн Чжан, Цзянь Хэ, Эшли М. Свинг и Кунь Ян

Аннотация

Справочная информация: распространённость японского шистосомоза значительно снизилась, а контроль в качестве средства борьбы с заболеванием уступил место его ликвидации провинции Цзянсу, КНР. Представляется важным и полезным исследование подхода к проведению оценки распространённости шистосомоза с использованием исключительно серологических данных.

Методы: мы собрали образцы сыворотки за период с 2011 по 2015 гг. с целью создания банка сыворотки по району Даньту, провинции Цзянсу, Китай. Образцы сыворотки были выявлены при помощи иммуноферментного твёрдофазного анализа (ELISA), были получены величины положительного коэффициента и оптической плотности (ОП). Была определена байесовская модель, включая предшествующую информацию о чувствительности и специфичности анализа ELISA, а также получена оценка уровня инфицирования по различным годам, половому признаку и возрастным группам.

Результаты: по среднему показателю ОП значительной разницы в зависимости от года и пола выявлено не было, однако наблюдалась значительная разница по возрастным группам. Наблюдались статистически значимые различия положительного коэффициента по различным годам и возрастным группам, при этом различия по половому признаку были незначительными. Расчётный уровень инфицирования за пять лет составил 1,288%, 1,456%, 1,032%, 1,485% и 1,358% соответственно. Не наблюдалось значительной разницы между различными годами и по половому признаку, однако присутствовала разница между различными возрастными группами.

Выводы: риск передачи шистосомоза по данному району до сих пор присутствует, следовательно, необходимо усилить отслеживание риска шистосомоза.

Translated from English version into Russian by Liudmila Tomanek and Galina Dmitrieva, through

Un enfoque serológico para estimar la prevalencia de la Schistosomiasis japonica en el condado de Dantu en Jiangsu, China.

Xin-Yao Wang, Jing Xu, Song Zhao, Wei Li, Jian-Feng Zhang, Jian He, Ashley M. Swing y Kun Yang.

Resumen

Introducción: la prevalencia de la *Schistosomiasis japonica* ha disminuido significativamente y las respuestas están cambiando de control a eliminación en la Provincia Jiangsu, China.La forma de estimar el cambio de prevalencia de esquistosomiasis usando sólo información serológica será importante y útil.

Metodología: Recolectamos muestras de suero de 2011 a 2015 para construir un banco de suero del

condado Dantu de Jiangsu, China. Las muestras de suero se analizaron con un ensayo por inmunoabsorción ligado a enzimas (ELISA por sus siglas en inglés) y se obtuvieron tasas de positividad y densidad óptica (DO). Se estableció un modelo bayesiano incluyendo la información previa sobre la sensibilidad y especificidad del ELISA, y se obtuvieron tasas estimadas de infección para diferentes años, géneros y grupos etarios.

Resultados: No hubo cambios significativos en las DO promedio entre diferentes años y géneros, pero hubo una diferencia significativa entre los diferentes grupos etarios. Hubo diferencias significativas en las tasas de positividad para diferentes años y grupos etarios pero no hubo diferencia significativa en los diferentes géneros. La tasa de infección estimada para los cinco años fue de 1,288%, 1,456%, 1,032%, 1,485% y 1,358%, respectivamente. No hubo diferencia significativa entre diferentes años y entre géneros, pero hubo una diferencia significativa entre los diferentes grupos etarios.

Conclusiones: El riesgo de transmisión de esquistosomiasis en esta área todavía existe y el monitoreo del riesgo de esquistosomiasis debería ser reforzado.

Translated from English version into Spanish by Alejandro PISCOYA and Emiliano S. Grill, through

TRANSLATORS
WITHOUT BORDERS
